# Development and validation of probe-based multiplex real-time PCR assays for the rapid and accurate detection of freshwater fish species

**DOI:** 10.1371/journal.pone.0210165

**Published:** 2019-01-30

**Authors:** Emily N. Hulley, Sujeenthar Tharmalingam, Andrew Zarnke, Douglas R. Boreham

**Affiliations:** 1 Laurentian University, Department of Biology, Ontario, Canada; 2 Northern Ontario School of Medicine, Medical Sciences Division, Ontario, Canada; University of Helsinki, FINLAND

## Abstract

Reliable species identification methods are important for industrial environmental monitoring programs. Probe based real-time quantitative polymerase chain reaction (qPCR) provides an accurate, cost-effective and high-throughput method for species identification. Here we present the development and validation of species-specific primers and probes for the *cytochrome c oxidase* (COI) gene for the identification of eight ecologically and economically important freshwater fish species: lake whitefish (*Coregonus clupeaformis*), yellow perch (*Perca flavescens*), rainbow smelt (*Osmerus mordax*), brook trout (*Salvelinus fontinalis*), smallmouth bass (*Micropterus dolomieu*), round whitefish (*Prosopium cylindraceum*), spottail shiner (*Notropis hudsonius*) and deepwater sculpin (*Myoxocephalus thompsonii*). In order to identify novel primer-probe sets with maximum species-specificity, two separate primer-probe design criteria were employed. Highest ranked primer-probe sets from both methods were assayed to identify sequences that demonstrated highest specificity. Specificity was determined using control species from same genus and non-target species from different genus. Selected primer-probe sets were optimized for annealing temperature and primer-probe concentrations to identify minimum reagent parameters. The selected primer-probe sets were highly sensitive, with DNA concentrations as low as 1 ng adequate for positive species identification. A decoder algorithm was developed based on the cumulative qPCR results that allowed for full automation of species identification. Blinded experiments revealed that the combination of the species-specific primer/probes sets with the automated species decoder resulted in target species identification with 100% accuracy. We also conducted a cost/time comparison analysis between the qPCR assays established in this study with other species identification methods. The qPCR technique was the most cost-effective and least time consuming method of species identification. In summary, probe-based multiplex qPCR assays provide a rapid and accurate method for freshwater fish species identification, and the methodology established in this study can be utilized for various other species identification initiatives.

## Introduction

Rapid and accurate species identification techniques are important for environmental monitoring programs, specifically in industrial settings. Large scale operations such as power plants, paper mills, petrochemical plants, petroleum refineries, natural gas and food processing plants negatively impact surrounding bodies of water due to use of once-through cooling systems. These system uptake large volumes of lake water where it absorbs heat from the condensers as it circulates throughout the plant before it is released back into the lake as a warmed surface effluent [1]. Surrounding wildlife become entrained or impinged at the intake sites of these systems [2]. Smaller fishes, such as embryo, larval and juveniles are more susceptible to these industrial processes because they have limited swimming capacity and therefore cannot avoid the water intake systems [2].

Morphological based species identification technique has often been the traditional method used by environmental monitoring programs to identify impinged fish species at intake points in once-through cooling operations. However, this technique has been proven to have several limitations, specifically with impinged specimens that are often damaged or missing key identification traits. Incorrect identification occurs due to intraspecific phenotypic plasticity of a trait or cryptic variation in both adult and larval fish stages [3–7]. In addition, key morphological traits for identification may only be effective for a particular gender or life stage. This is frequently an issue for larval fish identification as many species share similar morphology [3–6]. As well, species can rapidly change during development and often individuals from the same species can appear quite different [3–6]. Ko *et al*. 2013 compared the accuracy of morphological identification techniques of larval fish between five different laboratories and found the accuracy rates to be very low; 80.1% accuracy at the family-level, 41.1% accuracy for members among the same genus and 13.5% accuracy at the species-level. Consequently, a high level of expertise is required for morphological identification of larval fish, and often recommended that identification be conservative and limited to the family-level [4]. However, ecological and environmental studies require a high level of specificity and accuracy, requiring individuals to be identified to genus- and species-level [5].

Over the last decade, it has been shown that a 650-bp segment of the 5’ region of the mitochondrial *cytochrome c* oxidase I (COI) gene can serve as a universal barcode for the animal kingdom [3,5,7–9]. DNA barcoding identifies species through molecular tags based on short regions of DNA from standardised region of the genome (COI, *cytochrome b*, 16S ribosomal RNA, etc) [3,8]. DNA barcoding has proven to be a beneficial identification technique for the ecology of cryptic communities, forensic science, monitoring of invasive species and food traceability [3,7,8,10,11]. However, DNA barcoding requires several post-PCR processing steps (staining, gel electrophoresis, PCR purification, etc), which increases processing cost, time and the chance of product contamination.

Real-Time PCR (qPCR) coupled with hydrolysis probes provides an accurate and high-throughput alternative for species identification when compared to DNA barcoding [9,12–15]. The hydrolysis probe is dual-labelled with a fluorescent reporter dye (e.g FAM, VIC) and a light-absorbing quencher dye (e.g TAMRA, QSY). The probe is unable to emit fluorescent signal when the reporter and quencher dyes are in close proximity. During PCR amplification the probe is hydrolyzed during the extension phase by Taq DNA polymerase. This results in the separation of the dyes and an increase in reporter fluorescence [13,14,16]. When primers and probes are designed to be species-specific, fluorescence occurs only when the forward primer, reverse primer and probe hybridize to the target species DNA [13,16]. Here, the hydrolysis probe provides a further layer of specificity which is absent in SBYR Green PCR technology resulting in reduced false-positive amplifications [17]. Most importantly, probe-based qPCR provides the opportunity to process multiple samples in a single reaction (multiplex). Here, the hydrolysis probes can be linked to a variety of fluorescent dyes that have distinct excitation-emission spectra [9,12,13]. Indeed, probe-based qPCR assays have successfully been developed for numerous applications including detection of bacterial and pathogen strains [13,14], food traceability [9,15,18] and fish identification [12,16,19].

The objective of this study was to develop and validate species-specific probe-based qPCR assays for the identification of eight freshwater fish species for industrial environmental monitoring programs in the surrounding the Great Lakes region. The eight species of interest included lake whitefish (*Coregonus clupeaformis*), yellow perch (*Perca flavescens*), rainbow smelt (*Osmerus mordax*), brook trout (*Salvelinus fontinalis*), smallmouth bass (*Micropterus dolomieu*), round whitefish (*Prosopium cylindraceum*), spottail shiner (*Notropis hudsonius*) and deepwater sculpin (*Myoxocephalus thompsonii*). These fish are not only ecologically important for their surrounding ecosystems but many are of great economical importance for Canada’s commercial and recreational fishing industries. In 2005, it was estimated that commercial fisheries in the Great Lakes produced over 35 million dollars in Ontario alone [20]. Lake whitefish (*Coregonus clupeaformis*), yellow perch (*Perca flavenscens*) and rainbow smelt (*Osmerus mordax*) represent three of the top eight most harvested fish species in the Great Lakes with values of 8.4 million, 7.9 million and 1.1 million dollars (CAD) respectively [21]. Yellow perch had the greatest value per pound with a worth of over $2 per pound [21]. Lake whitefish is also an important sustenance fishery for the surrounding Aboriginal communities. Sport fishing is another multibillion dollar industry that relies heavily on the productivity of the Great Lakes. The Great Lakes recreational fishing has an estimated annual net value range of 393 million to 1.47 billion dollars [22]. Furthermore, yellow perch, smallmouth bass (*Micopterus dolomieu*) and brook trout (*Salvelinus fontinalis*) are popular fish species targeted by anglers in the Great Lakes [23]. Studies have estimated that anglers pay 40 to 55 dollars per day for these species [22]. Although the round whitefish and deepwater sculpin are not economically important, these species were included in this study since round whitefish is highly sensitive to nuclear power plant operations [24] and deepwater sculpin is an endangered species [25].

Utilizing the DNA barcoding gene, COI, species-specific primers and hydrolysis probes were developed for the eight species of interest. In order to identify novel primer-probe sets with maximum species-specificity, two separate primer-probe design criteria were employed. Highest ranked primer-probe sets from both methods were assayed to identify sequences that demonstrated highest specificity. Specificity was determined using control species from same genus and non-target species from different genus. Temperature, primer and probe concentrations were optimized for selected primer-probe sets before they were comprehensively validated through numerous qPCR reactions. A species decoder algorithm was developed based on the qPCR results to automatically identify species thereby allowing full automation of results analysis. Blinded experiments were performed to test the accuracy of the automated species decoder. Finally, a cost/time comparison analysis was performed between the qPCR assays established in this study with other species identification methods.

## Materials and methods

### Sample collection & DNA extraction

Morphologically identified samples were provided from several external sources outlined in S1 Table. DNA was extracted from individual fish muscle tissue, fin clips or liver samples in 10mm samples sizes (S1 Table). Extractions were performed using the Qiagen DNEasy Blood and Tissue Kit (Mississuaga, ON) following manufactures guidelines. DNA was eluted from the columns using MilliQ grade nuclease-free water. DNA concentrations, 260:280 and 260:230 ratios were measured using a NanoDrop 1000 spectrophotometer (Thermo Scientific). Samples were included only when 260:280 and 260:230 ratios were between 1.8–2.0 and 2.0 to 2.2 respectively.

### DNA barcoding

Extracted DNA was diluted to 35ng/μL. Polymerase chain reactions (PCR) were run using the universal fish primers Fish F1 (5’-TCA ACC AAC CAC AAA GAC ATT GCC AC-3’) and Fish R1 (5’-TAG ACT TCT GGG TGG CCA AAG AAT CA-3’) which amplified a 658 base pair (bp) region of the COI gene [26]. PCR reactions consisted of a total volume of 25 μL with 12.5 μL of iQ Supermix (BioRad Laboratories, Mississauga, ON), 0.25 μL of 100 μM of each primer and 7 ng of template DNA. PCR reactions were run on MJ Mini Personal Thermal Cycler (BioRad Laboratories, ON) with thermal cycling regime of 2 minutes at 94°C, 35 cycles of 30 seconds at 94°C, 40 seconds at 52°C, 1 minute at 72°C and a final extension of 72°C for 10 minutes. All PCR products were verified on a 1% agrose gel (BioRad Laboratories, ON).

For further PCR product sequencing the PCR products were purified using the Qiagen MinElute PCR Purification Kit (Mississauga, ON) following manufactures guidelines. Final elutions were carried out using MilliQ grade nuclease-free water with final elution volume of 10 μL. 0.7 μL of 5 pmol of forward primer was added to 7 μL of purified product. All samples were sequenced using the Sanger method at The Centre for Applied Genomics at SickKids (Toronto, ON).

Specimens were identified by Blast analysis comparing the percentage of homology between COI sequenced amplicons and the sequences available at the NCBI database (http://blast.ncbi.nlm.nih.gov/Blast.cgi). Species were considered a match when there was ≥98% similarity to an individual species coupled with a bit score of ≥1000.

### Sequence alignment

COI sequences were collected for the target species and control species from the same genus (CON) from Barcode of Life Database (BOLD) (http://www.boldsystems.org/). The sequences were aligned using a multiple sequence alignment software (T-Coffee) (https://www.ebi.ac.uk/Tools/msa/tcoffee/). The selected primer-probe sets were analyzed to ensure that there were adequate mismatches between the primer-probe sequences and the corresponding regions on the COI gene of the CONs. Mismatches were calculated as number of bp differences between the CON sequences and the primer-probe sequences. Bp differences were highlighted and summed for each primer-probe set.

### Design of primer and probe

Utilizing BOLD, all available sequences for each of the eight target species were obtained regardless of geographical location in order to avoid intraspecies variation: 260 for lake whitefish, 47 for smallmouth bass, 15 for deepwater sculpin, 61 for spottail shiner, 118 for rainbow smelt, 72 for yellow perch, 30 for round whitefish and 46 for brook trout. COI sequences were also collected for closely related species of each target species (CON). This included all species within the same genus of the target that are found in Canada (Table 1).

**Table 1:** COI gene sequence homology analysis of species of interest and control species from the same genus (CON).

Species of Interest	Control Species (same genus as species of interest)	Present in Great Lakes (Y/N)*	COI Gene Homology (Control Species/Species of Interest)	
			Base Pair Match	Percent (%)
**Lake Whitefish** **(*Coregonus clupeaformis*)**	**Cisco (*C*. *artedi*)**	Y	638/652	98%
	**Bloater (*C*. *hoyi*)**	Y	638/652	98%
	**Kiyi (*C*. *kiyi*)**	Y	636/652	98%
	Blackfin Cisco (*C*. *nigripinnis*)	Y	634/652	97%
	Shortjaw Cisco (*C*. *zenithicus*)	Y	636/652	98%
	Arctic Cisco (*C*. *autumnalis*)	N	639/652	98%
	Atlantic Whitefish (*C*. *huntsmani*)	N	632/652	97%
	Bering Cisco (*C*. *laurettae*)	N	637/652	98%
	Broad Whitefish (*C*. *nasus*)	N	645/652	99%
	Humpback Whitefish (*C*. *pidschian*)	N	645/648	99%
	Sardine Cisco (*C*. *sardinella*)	N	640/652	98%
**Smallmouth Bass (*Micropterus dolomieu)***	**Largemouth Bass (*M*. *salmoides*)**	Y	600/652	92%
**Deepwater Sculpin (*Myoxocephalus thompsonii*)**	Fourhorn Sculpin (*M*. *quadricornis*)	N	649/652	99%
**Spottail Shiner** **(*Notropis hudsonius*)**	**Pugnose Shiner (*N*. *anogenus*)**	Y	586/648	90%
	Emerald Shiner (*N*. *atherinoides*)	Y	590/651	91%
	Bridle Shiner (*N*. *bifrenatus*)	Y	579/651	89%
	Ghost Shiner (*N*. *buchanani*)	Y	594/651	91%
	**Blackchin Shiner (*N*. *heterodon*)**	Y	585/648	90%
	Blacknose Shiner (*N*. *heterolepis*)	Y	593/651	91%
	**Silver Shiner (*N*. *photogenis*)**	Y	590/651	91%
	**Rosyface Shiner (*N*. *rubellus*)**	Y	585/651	90%
	**Sand Shiner (*N*. *stramineus*)**	Y	571/645	89%
	**Weed Shiner (*N*. *texanus*)**	Y	570/651	88%
	**Mimic Shiner (*N*. *volucellus*)**	Y	590/651	91%
	**Bigmouth Shiner (*N*. *dorsalis*)**	N	586/651	90%
	**River Shiner (*N*. *blennius*)**	N	579/642	90%
	**Carmine Shiner (*N*. *percobromus*)**	N	592/651	91%
**Rainbow Smelt** **(*Osmerus mordax*)**	Pacific Rainbow Smelt (*O*.*dentex*)	N	597/648	92%
**Yellow Perch** **(*Perca flavescens)***	Logperch (*Percina caprodes*)	Y	643/652	83%
	**Channel Darter (*Percina copelandi*)**	**Y**	**557/652**	**85%**
	**Blackside Darter (*Percina maculata*)**	**Y**	**553/653**	**85%**
	River Darter (*Percina shumardi*)	Y	551/654	84%
**Round Whitefish (*Prosopium Cylindraceum*)**	**Pygmy Whitefish (*P*. *coulterii*)**	Y	583/651	90%
	Mountain Whitefish (*P*. *williamsoni*)	N	624/651	96%
**Brook Trout** **(*Salvelinus fontinalis*)**	**Bull Trout (*S*. *confluentus*)**	Y	611/652	94%
	**Lake Trout (*S*. *namaycush*)**	Y	613/652	94%
	**Arctic Char (*S*. *alpinus*)**	N	616/652	94%
	**Dolly Varden (*S*. *malma*)**	N	617/652	95%

Homology score is represented as the number of base pair matches in the COI gene of control species compared to species of interest. Bolded names indicate control species used as negative controls in validating the primer-probe sets.

*FishBase 2018

Primer-BLAST software offered by National Center for Biotechnology Information (NCBI) (https://www.ncbi.nlm.nih.gov/tools/primer-blast/) was utilized to obtain PCR primer-probe sequences for the species specific COI gene. The following parameters were applied to the software for designing forward and reverse primer pairs: minimum and maximum PCR product size of 70 to 150 respectively, minimum and maximum primer melting temperature of 57 to 63°C with a maximum temperature difference of 3°C between forward and reverse primer sets. As well, corresponding probe parameters included: minimum and maximum size of 18 and 27 nucleotides respectively and a minimum and maximum primer melting temperature of 57 and 63°C.

Primer pair specificity was determined using two different methods:

#### Method #1

Primer-BLAST was utilized to obtain the top 10 forward and reverse primer pairs and corresponding probe sequences for the COI gene for each species of interest. Species specificity was determined using COI gene sequences from relevant CONs within the Great Lakes. Each primer set (forward, reverse and probe) identified by Primer-BLAST was compared to each CON COI sequence. Primers that had exact matching sequences were given a score of 1. For example, if both the forward primer and probe had exact complementary binding to a CON gene then a score of 2 was given. This analysis was performed for all relevant CON sequences for all 10 primer sets. Scores from all CON COI sequences were summed and the primer sets that scored zero or lowest were considered species specific. When multiple primer sets scored zero, the primer set with the highest rank from Primer-BLAST was chosen for qPCR analysis.

#### Method #2

A phylogenetic tree for COI gene sequences for relevant CONs and species of interest were generated using a multiple sequence alignment software (T-Coffee). The phyogenetic cladogram revealed the CON species that were most homologous to the species of interest. The COI sequence for the most closely related CON was inputted in the primer specificity component of Primer-BLAST software. This feature allowed Primer-BLAST to avoid regions that are shared between the species of interest and the most closely related CON. The top ranking set (forward, reverse and probe) identified by Primer-BLAST by this method was chosen for qPCR analysis.

#### Selection of primer-probe sequences from method #1 and method#2

Top ranking primer-probe sequences obtained from Methods #1 and #2 were screened using SYBR green based qPCR analysis to identify the forward and reverse primers that demonstrated the lowest C_q_ (quantification cycle) value. For this initial screen, SYBR green methodology was chosen over hydrolysis technology since we wanted to ensure that the forward and reverse sequences were species-specific even without considering the additional specificity obtained using the hybridization probe. Additionally, SYBR green qPCR allows us to screen Methods #1 and #2 without having to purchase expensive hydrolysis probes.

### qPCR

Selected primers were purchased from Sigma-Aldrich Canada Co. (Oakville, ON). Optimal primer annealing temperature and species specificity were validated using QuantStudio5 Real-Time PCR System (Applied Biosystems by Thermo Fisher, ON). SYBR-green based qPCR reactions were prepared in 15 μL volumes containing 2X SensiFAST Sybr Lo-Rox Mix (Bioline, Boston, MA), 300 nM of forward and reverse primer, diethyl pyrocarbonate (DEPC) water and 100 ng of DNA. qPCR reactions were performed in duplicate using the parameters of (1) 95°C for 2 minutes, (2) 95°C for 10 seconds, followed by (3) 58°C for 10 seconds and (4) 72°C for 20 seconds. Steps 2–4 were repeated for 40 cycles followed by a melt curve analysis.

Hydrolysis probes from selected primer sets were ordered from Applied Biosystems, Thermo Fisher Scientific (Foster City, CA). Fluorescents dyes chosen for the hydrolysis probers were FAM (~517nm), VIC (~551nm), ABY (~580nm) and JUN (~617nm). Probes were purchased with QSY quenchers and HPLC purified in unit size of 6000 pmol (1xTE/100pmol format). Primers and hydrolysis probe optimization and validation were performed using QuantStudio5 Real-Time PCR System. hydrolysis probe-based qPCR reactions were prepared in 20 μL volumes containing 100ng of DNA, 50nM to 500nM of forward and reverse primers, 100nM to 200nM of hydrolysis probe, 10 μL hydrolysis Multiplex Master Mix (Applied Biosystems, CA) and DEPC water. Reactions were run in MicroAmp Optical 96-well reaction plates (Applied Biosystems, CA) using the parameters of 95°C for 20 seconds, followed by 95°C for 1 seconds, 60°C for 20 seconds. This was repeated for 40 cycles. Optimal primer annealing temperature was obtained by performing a temperature gradient of 54°C to 64°C during the annealing stage. Primers and probe concentrations were also extensively optimized. Forward and reverse primers were prepared in varying concentrations of 50, 100, 200 and 400nM and hydrolysis probes in concentrations of 100, 200 and 250nM. Optimal primer and probe concentrations were determined based on amplification with the lowest C_q_ value.

Hydrolysis probe sets were validated through single and multiplexing reactions with DNA from species of interest and their corresponding control species (same genus) or non-target species (different genus). Single-plexing reactions had 100 ng of DNA from a single species of interest and the corresponding primer-probe set. Multiplexing reaction had four primer-probe sets multiplexed together in a single well with either single or multiple species of DNA. Species detection limits and specificity were validated through 10-fold 8-point standard curves with DNA concentrations of 0.001ng to 300ng. All validation reactions were run in duplicate.

### Automated data decoder algorithm

A species decoder algorithm was developed to automatically analyze the qPCR data for full automation of species identification. In order to standardize DNA input, 100 ng of sample DNA was chosen for all reactions. Based on the cumulative qPCR results, the following parameters obtained from the QuantStudio software (ThermoFisher) were incorporated into the algorithm. The first parameter included a species-specific C_q_ cut-off value of 25; detection of all target species occurs below this value and any false detection of non-target species is well above this value (see Results). Next we utilized the “amplification status” parameter obtained from the QuantStudio software. In certain qPCR reactions, phantom signals are inappropriately associated with a C_q_ value. The QuantStudio software utilizes proprietary algorithms and determines whether this amplification is true or false. Therefore, only true amplification reactions as determined by QuantStudio software were included in the analysis. Finally, the “normalized reporter value” (ΔRn) of greater than 0.3 was the final parameter included in the algorithm. ΔRn is also used to avoid phantom signals and is calculated by the magnitude of fluorescent signal over the background noise of the dye. Therefore, if a signal had positive amplification status and ΔRn value of 0.3 or greater the algorithm deemed the signal as a positive amplification.

## Results

### Species of interest and establishment of control species using geographical and homology analysis

In order to determine primer-probe specificity, we established a list of control species that were highly homologous to the species of interest (Table 1). This was achieved by obtaining COI gene sequence information for the target species and their respective CONs using BOLD and FishBase (http://www.fishbase.org/). Table 1 reports the alignment scores as percent homology of CON species versus target species and lists the CON species based on homology rank. As well, the location of each CON habitat with regards to the Great Lakes was reported in Table 1. This was performed to discredit certain highly homologous CON species when not geographically present in the Great Lakes.

Table 1 demonstrates that many of the most closely related CONs were not located within the Great Lakes, specifically fourhorn sculpin, broad and humpback whitefish which had 99% homology match to their respective target species. *Notropis* and *Percina* were the exception with their most closely related CONs located in the Great Lakes. Nonetheless, percent homologies for these CONs were only 91% and 85% respectively, which allows for adequate gene sequence dissimilarity for the design of target-specific primer-probe sets. *Coregonus* was the only genus to exhibit CONs within the Great Lakes that had percent homology greater than 96%. All other target species had CONs within the Great Lakes with sequence homology below 94%. Therefore, species of interest had either geographical separation from its most closely related CON or the most closely related CON had a lower than 94% homology, with the Coregonus genus being the exception. Selected CON species utilized as negative controls for the validation of the primer-probe sets are bolded in Table 1.

To ensure samples used in this study were correctly identified a subset of the target and non-target samples were barcoded with the COI gene and sequenced using the Sanger method. All sequences had ≥98% homology to their corresponding species, which were previously identified by morphological parameters.

### Selection of primer-probe sequences

Primer-probe sequences were designed using two novel approaches as described in the *Materials and Methods* section. Analysis of forward and reverse primer specificity using SYBR green based qPCR analysis with 100 ng of the appropriate DNA sample is shown in S2 Table. Overall, C_q_ values ranged between 12–19 and all reactions demonstrated single melt-curve peaks. Interestingly, C_q_ values obtained from Methods #1 and #2 were within one C_q_ value demonstrating that both methods provided primer sequences with similar specificity. Nonetheless, the design methodology that resulted with the lowest C_q_ value was chosen. Therefore, primer-probe sequences for yellow perch and brook trout were obtained from Method #2, while Method #1 was utilized the remaining six species (Table 2).

**Table 2:** Selected primert and probe sequences targeting the COI gene.

Target Species	Fluorescent Dye	Primer/ Probe	Sequence	Optimal Concentrations (nM)	Optimal Temperature (°C)	Amplicon Size
Smallmouth Bass	FAM	Forward	TCTTTCCTTCTCCTGCTCGC	50	60	147
		Reverse	GGAGACACCCGCAAGATGAA	50		
		Probe	GCTGGAGCTGGCACTGGGTG	100		
Spottail Shiner	VIC	Forward	CTATTATTAGCTTCTTCTGGGGTTG	50	60	105
		Reverse	GAGGTCTACTGATGCGCCC	50		
		Probe	GCAGGCAATCTTGCCCACGC	100		
Round Whitefish	ABY	Forward	AATGTAATCGTCACGGCCCA	500	60	125
		Reverse	CGGGGGAATGCTATATCGGG	500		
		Probe	TGACTAATTCCCCTTATGATCGGAGCA	100		
Brook Trout	JUN	Forward	CGGTACGGGGTGAACAGTTT	400	60	103
		Reverse	GGAAATGCCAGCTAAATGTAGGG	400		
		Probe	CTCGCCCACGCAGGAGCTTC	200		
Lake Whitefish	FAM	Forward	TCTCCCTCCACTTAGCTGGT	200	60	118
		Reverse	GCCCAGACAAAAAGAGGGGT	400		
		Probe	TTCCTCTATCTTGGGGGCCGTT	200		
Deepwater Sculpin	VIC	Forward	CTTAGCCTCTTCGGGGGTTG	100	60	148
		Reverse	TGCTCCGAGGATCGAAGAGA	100		
		Probe	CCACGCGGGAGCCTCTGTTG	100		
Rainbow Smelt	ABY	Forward	CGATTATGATCGGCGGGTTTG	400	60	76
		Reverse	ATGCGAGGGAAGGCCATATC	400		
		Probe	CCCCCTTATGATTGGGGCCCCA	200		
Yellow Perch	JUN	Forward	GATCGGTGCCCCTGACATAG	200	60	146
		Reverse	TCCCAGCAAGAGGGGGATAA	400		
		Probe	AAGCCGGAGCTGGTACCGGA	200		

### *In Silico* verification of primer-probe specificity

Due to the high degree of homology between species of interest and their respective CONs (Table 1), the selected primer-probe sets were analyzed to ensure that there were adequate mismatches between the primer-probe sequences and the corresponding regions on the COI gene of the CONs. To accomplish this, COI sequences for target species and their respective CONs were aligned against the selected primer and probe sequences. Number of bp mismatches from the CON sequences and the primer-probe sequences were summed and highlighted in S3 Table. These alignments revealed that all eight primer-probe sets demonstrated perfect alignment with the corresponding target sequences while numerous mismatches were evident when aligned with the CON sequences or sequences from non-target species. In fact, the majority of the bp differences among non-target species ranged from 10 to 19. In regards to the alignment of the primer-probe sequences with corresponding CON, brook trout, yellow perch, rainbow smelt, spottail shiner, and round whitefish all had bp differences of 6 or greater. Smallmouth bass CON, largemouth bass scored lower with bp differences of 5. Deepwater sculpin and lake whitefish scored the lowest for number of mismatches between their CONs and primer-probe sequences (S3 Table). Fourhorn sculpin (deepwater sculpin CON), broad whitefish and sardine cisco (lake whitefish CONs) scored the lowest with bp differences of 1, 1 and 2 respectively. However, these three species are not located within the Great Lakes (Table 1). More importantly, the majority of the CONs for lake whitefish that are located within the Great Lakes demonstrated differences of 3 to 5bps. It is important to note that though the existence of only few differences among the sequences, they are spread over the reverse primer and probe sequences, increasing specificity for the primer-probe set. Therefore based on the high number of bp differences between the primer-probe sequences and the corresponding CON regions, this analysis revealed that the selected primer-probe sets target COI regions that are highly species specific.

### Probe specification and primer-probe qPCR optimizations

The eight primer-probes were randomly grouped into two sets of four primers to maximize the multiplexing capacity of the qPCR machine while minimizing costs required for reagents. The first set and their corresponding fluorescent dyes included smallmouth bass (FAM), spottail shiner (VIC), round whitefish (ABY) and brook trout (JUN), and the second set comprised of lake whitefish (FAM), deepwater sculpin (VIC), rainbow smelt (ABY) and yellow perch (JUN).

Temperature optimization experiments under single-plex conditions revealed that the primer-probe sets amplified target regions with equal efficiencies at annealing temperatures between 54–64°C; however 60°C was chosen as the optimal temperature for all primer-probe sets based on the manufacture’s recommended temperature for the QSY quenchers. Concentration optimization runs established that the primer-probe sets for deepwater sculpin, smallmouth bass and spottail shiner performed most efficiently at lower concentrations ranging from 50 and 100nM. Whereas round whitefish, brook trout, and rainbow smelt had higher optimal concentrations for their primer sets ranging from 400 and 500nM. Table 2 lists the primer/probe sequences (5’ to 3’), the respective fluorescent probe dye, optimal primer/probe concentrations and annealing temperature, and the resulting amplicon size.

The table lists the primer/probe sequences (5’ to 3’) for qPCR analysis, the respective fluorescent probe dye, optimal primer/probe concentrations and annealing temperature, and the resulting amplicon size.

### Determination of primer-probe sensitivity under single-plex vs multiplex qPCR conditions

**Fig 1:**
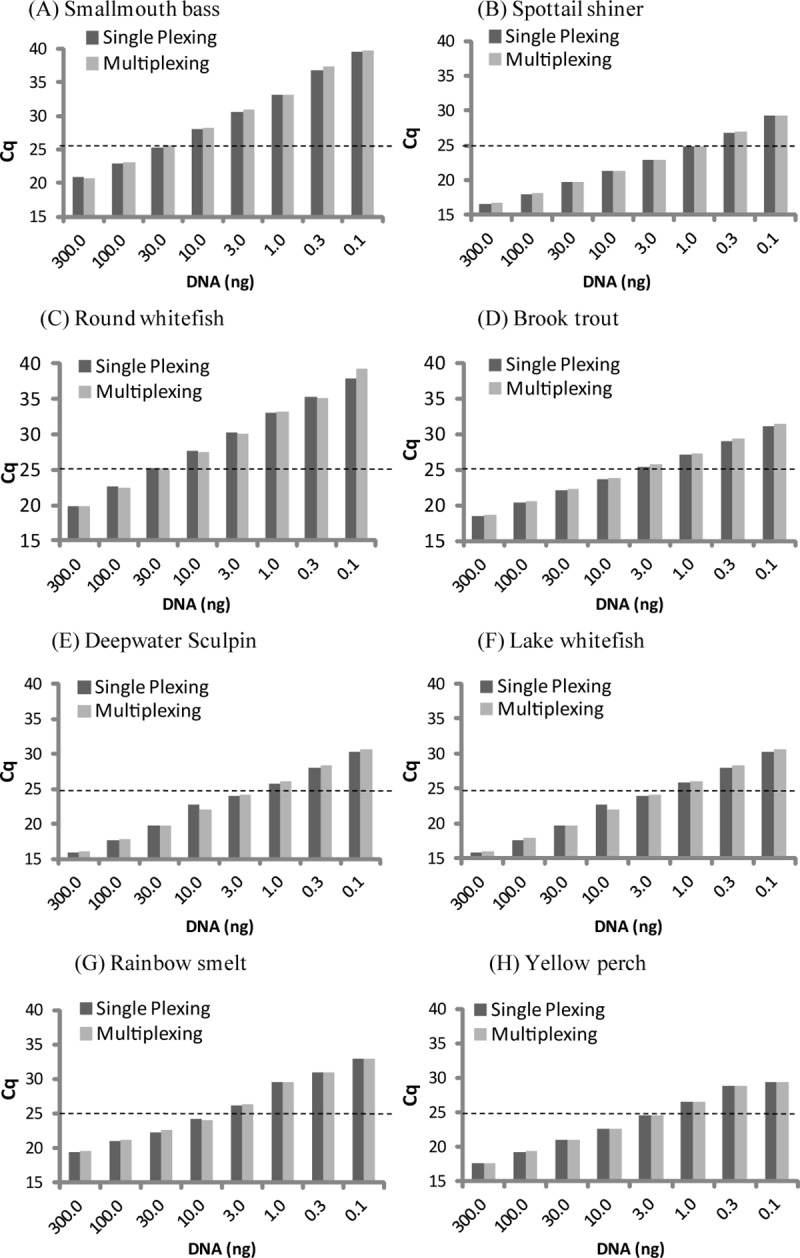
DNA concentration versus qPCR C_q_ values for each of the species-specific primers/probe sets. Data represents DNA concentrations from 0.03 ng to 300 ng under single-plex and multiplex qPCR conditions. Sensitivity of primer-probe set highlighted by the species-specific C_q_ cut-off value of 25 (dotted line).

In order to establish the DNA detection limits of the primer-probe sets, single-plex qPCR reactions were performed using target species DNA concentrations ranging from 0.001ng to 300ng. All primer-probe sets were able to amplify their target species at very low concentrations (Fig 1 and S4 Table). In detail, primer-probe sets for spottail shiner, brook trout, lake whitefish, yellow perch and deepwater sculpin were able to detect the respective target species at DNA concentrations as low as 0.001ng. Rainbow smelt primers-probe demonstrated amplification at DNA concentrations of 0.003ng, while smallmouth bass and spottail shiners were effective as low as 0.1ng DNA. Taken together, the primer-probe sets demonstrated excellent detection limits revealing that the sets are highly sensitive.

To determine the effects of multiplex analysis on the sensitivity of the primer-probe sets, qPCR runs were performed using target species DNA (0.001ng to 300ng) under multiplex conditions. Fig 1 demonstrates that all primer-probe sets demonstrated similar detection limits under both single-plex and multiplex conditions (S4 Table). The C_q_ values for multiplexing were within 0.1 to 1.3 when compared to their respective single-plex reactions. Therefore, these results demonstrate that the primer-probe set combinations are multiplex compatible.

### Determination of primer-probe specificity

The specificity of the primer-probe sets were established by performing qPCR reactions using DNA (0.001ng to 300ng) from appropriate CON species (S4 Table). These results demonstrated that the most closely related CONs for smallmouth bass and lake whitefish were undetectable at all concentrations. CONs for round whitefish, yellow perch, spottail shiner and brook trout were only detectable at higher DNA concentrations of 300 and/or 100ng, with significantly higher C_q_ values than the targets (35.48 to 39.7 for CONs compared to below 20 for target species) (S4 Table). Taken together, the primer-probes sets are ineffective in amplifying CON sequences demonstrating that the sets are highly specific for the intended target species.

A rigorous specificity analysis was conducted by performing all possible combinations of primer-probe sets with 100 ng DNA from species of interest, CONs or non-target species (different genus). At 100 ng, the primer-probes robustly detected their respective target species with C_q_ values between 16.21±0.35 and 23.79±0.69 (Table 3). More importantly, the majority of CON species and non-target species were undetectable, while others were detected with C_q_ values greater than 32. These results establish that at 100 ng DNA, target species can be detected with C_q_ value below 25. Therefore by setting a C_q_ cut-off value of 25 or below, these results suggest that target species can be identified with 100% accuracy and complete absence of false-positive detection.

**Table 3:** qPCR C_q_ values for species of interest, control species (same genus) and non-target species (different genus) for each species-specific primers/probe set.

Probe Set 1				Probe Set 2			
Primer/ Probe Set	Species	# of Samples	Average C_q_ Value	Primer/ Probe Set	Species	# of Samples	Average C_q_ Value
**Smallmouth Bass**	Smallmouth Bass	10	23.79 ± 0.69	**Lake Whitefish**	Lake Whitefish	5	16.21 ± 0.35
	Spottail Shiner	10	-		Deepwater Sculpin	5	-
	Round Whitefish	10	-		Rainbow Smelt	14	-
	Brook Trout	4	-		Yellow Perch	10	-
	Lake Whitefish	5	-		Smallmouth Bass	10	37.66 ± 0.21
	Deepwater Sculpin	5	-		Round Whitefish	10	37.71 ± 0.11
	Rainbow Smelt	14	-		Spottail Shiner	10	-
	Yellow Perch	10	-		Brook Trout	4	-
	Largemouth Bass	6	-		Cisco	2	-
**Spottail Shiner**	Spottail Shiner	10	22.61 ± 1.14		Bloater	1	-
	Smallmouth Bass	10	-		Kiyi	1	-
	Round Whitefish	10	-	**Deepwater Sculpin**	Deepwater Sculpin	5	18.13 ± 0.43
	Brook Trout	4	-		Lake Whitefish	5	-
	Lake Whitefish	5	-		Rainbow Smelt	14	-
	Deepwater Sculpin	5	-		Yellow Perch	10	-
	Rainbow Smelt	14	-		Smallmouth Bass	10	-
	Yellow Perch	10	-		Round Whitefish	10	-
	Bigmouth Shiner	2	-		Spottail Shiner	10	-
	Carmine Shiner	3	-		Brook Trout	4	-
	Rosyface Shiner	2	-	**Rainbow Smelt**	Rainbow Smelt	14	18.83 ± 0.38
	Weed Shiner	3	-		Lake Whitefish	5	-
**Round Whitefish**	Round Whitefish	10	19.79 ± 1.18		Deepwater Sculpin	5	-
	Smallmouth Bass	10	-		Yellow Perch	10	-
	Spottail Shiner	10	-		Smallmouth Bass	10	-
	Brook Trout	4	-		Round Whitefish	10	35.83 ± 0.49
	Lake Whitefish	5	-		Spottail Shiner	10	-
	Deepwater Sculpin	5	-		Brook Trout	4	32.66±0.039
	Rainbow Smelt	14	-	**Yellow Perch**	Yellow Perch	10	19.12 ± 0.37
	Yellow Perch	10	36.35 ± 0.46		Lake Whitefish		-
	Pygmy Whitefish	3	35.5 ± 0.43		Deepwater Sculpin		
**Brook Trout**	Brook Trout	4	16.74 ± 0.65		Rainbow Smelt		-
	Smallmouth Bass	10	-		Smallmouth Bass		-
	Spottail Shiner	10	-		Round Whitefish		36.36 ± 0.46
	Round Whitefish	10	-		Spottail Shiner		35.36 ± 0.91
	Lake Whitefish	5	32.61 ± 0.15		Brook Trout		-
	Deepwater Sculpin	5	-		Log Perch	3	-
	Rainbow Smelt	14	-		Blackside Darter	2	39.04 ± 1.96
	Yellow Perch	10	-		River Darter	2	-
	Lake Trout	2	39.64 ± 1.36				
	Arctic Char	2	38.49 ± 0.19				
	Bull Trout	2	36.74 ± 0.52				
	Dolly Varden	2	39.36 ± 0.25				

100ng of DNA was used in all reactions. Species that were undetectable are marked as “-”.

### Blinded analysis utilizing automated data decoder algorithm

A species decoder algorithm was developed to automatically analyze the qPCR data for full automation of species identification (parameters described in *Materials and Methods*). To ensure reliability and accuracy of the automated species decoder algorithm and the primer-probe sets, a comprehensive blinded study was performed that included all species of interest and their corresponding CONs. Table 4 shows that all species of interest were successfully identified by their corresponding primer-probe sets when performed in a blinded fashion (Table 4). Furthermore, the species decoder algorithm had zero percent false positive detection of CON and non-target species (Table 4). As a whole, the blinded experiments revealed that the combination of the species-specific primer/probes sets with the automated species decoder resulted in target species identification with 100% accuracy.

**Table 4:** Blinded experiments revealed that the species-specific primer/probe sets identified target species with 100% accuracy.

Probe Set 1				Probe Set 2			
Primer Set	Species	# of samples	# of positive IDs	Primer Set	Species	# of samples	# of positive IDs
**Smallmouth Bass**	Smallmouth Bass	10	10	**Lake Whitefish**	Lake Whitefish	5	5
	Largemouth Bass	6	0		Cisco	2	0
	Spottail Shiner	10	0		Bloater	1	0
	Spottail Shiner CONs	14	0		Kiyi	1	0
	Round Whitefish	11	0		Blackfin Cisco	1	0
	Pygmy Whitefish	3	0		Deepwater Sculpin	5	0
	Brook Trout	4	0		Fourhorn Sculpin	1	0
	Brook Trout CONs	8	0		Rainbow Smelt	14	0
**Spottail Shiner**	Spottail Shiner	10	10		Yellow Perch	10	0
	Weed Shiner	3	0		Yellow Perch CONs	7	0
	Carmine Shiner	3	0	**Deepwater Sculpin**	Deepwater Sculpin	5	5
	Rosyface Shiner	2	0		Fourhorn Sculpin	1	0
	Bigmouth Shiner	2	0		Lake Whitefish	5	0
	Mimic Shiner	1	0		Lake Whitefish CONs	5	0
	Silver Shiner	1	0		Rainbow Smelt	14	0
	Sand Shiner	1	0		Yellow Perch	10	0
	Blackchin Shiner	1	0		Yellow Perch CONs	7	0
	Pugnose Shiner	1	0	**Rainbow Smelt**	Rainbow Smelt	14	14
	River Shiner	1	0		Deepwater Sculpin	5	0
	Smallmouth Bass	10	0		Fourhorn Sculpin	0	0
	Largemouth Bass	6	0		Lake Whitefish	5	0
	Round Whitefish	11	0		Lake Whitefish CONs	5	0
	Pygmy Whitefish	3	0		Yellow Perch	10	0
	Brook Trout	4	0		Yellow Perch CONs	7	0
	Brook Trout CONs	8	0	**Yellow Perch**	Yellow Perch	10	10
**Round Whitefish**	Round Whitefish	11	11		Log Perch	3	0
	Pygmy Whitefish	3	0		Blackside Darter	2	0
	Smallmouth Bass	10	0		River Darter	2	0
	Largemouth Bass	6	0		Rainbow Smelt	14	0
	Spottail Shiner	10	0		Deepwater Sculpin	5	0
	Spottail Shiner CONs	14	0		Fourhorn Sculpin	0	0
	Brook Trout	4	0		Lake Whitefish	5	0
	Brook Trout CONs	8	0		Lake Whitefish CONs	5	0
**Brook Trout**	Brook Trout	4	4				
	Arctic Char	2	0				
	Bull Trout	2	0				
	Lake Trout	2	0				
	Dolly Varden	2	0				
	Smallmouth Bass	10	0				
	Largemouth Bass	6	0				
	Round Whitefish	11	0				
	Pygmy Whitefish	3	0				
	Spottail Shiner	10	0				
	Spottail Shiner CONs	14	0				

A comprehensive blinded study was performed using the species-specific primer-probe sets in combination with randomized samples consisting of species of interest, control species (same genus) and non-target species (different genus). An automated species decoder algorithm was employed for species identification based on qPCR values. The algorithm utilized species-specific C_q_ value cut-off of 25 and a positive amplification signal with a normalized reporter value (ΔRn) greater than 0.3. All qPCR reactions used 100 ng of sample DNA.

### Multiplexing of multiple species DNA

For further validation and economical purposes we were interested in determining whether the primer-probe sets were able to identify their corresponding species of interest when multiple different species DNA was present in a single reaction. When multiplexed with DNA from multiple different species in a single well, all species of interest were correctly identified by their corresponding primer-probe sets (Table 5). DNA detection limit analysis showed that spottail shiner and deepwater sculpin were detected with as low as 0.001ng of DNA. The majority of primer sets detected their corresponding target species to concentrations of 0.03ng excluding smallmouth bass and round whitefish which detected to 1.0ng and 0.1ng respectively. At a 100ng DNA input, spottail shiner, brook trout, lake whitefish, deepwater sculpin, rainbow smelt and yellow perch primer-probe sets were able to detect their corresponding target DNA within the species decoder C_q_ cut-off of 25 (Table 5). Round whitefish and smallmouth bass however had C_q_ values slightly above this cut-off with values of 25.23±0.22 and 28.71±0.058 respectively. Therefore, if multiple species samples were mixed and unable to be separated, species can be identified using the species decoder; however C_q_ cut-off values would need to be adjusted to account for smallmouth bass and round whitefish primer-probe sets. Taken at large, the overall results demonstrate that the probe-based multiplex qPCR assays developed in this study are highly sensitive and robustly accurate.

**Table 5:** Primer/probe sets specifically amplify target species despite presence of DNA from multiple species.

Probe Set 1			Probe Set 2		
Probe Set	Species Sample	Ct	Probe Set	Species Sample	Ct
**Smallmouth Bass**	Smallmouth bass	28.71 ± 0.058	**Lake Whitefish**	Lake Whitefish	21.48 ± 0.065
	Spottail shiner	undetectable		Deepwater Sculpin	undetectable
	Round whitefish	undetectable		Rainbow Smelt	undetectable
	Brook Trout	undetectable		Yellow Perch	undetectable
**Spottail Shiner**	Spottail shiner	21.37 ± 0.15	**Deepwater Sculpin**	Deepwater Sculpin	21.37 ± 0.027
	Smallmouth bass	undetectable		Lake Whitefish	undetectable
	Round whitefish	undetectable		Rainbow Smelt	undetectable
	Brook Trout	undetectable		Yellow Perch	undetectable
**Round Whitefish**	Round whitefish	25.23 ± 0.22	**Rainbow Smelt**	Rainbow Smelt	21.48 ± 0.048
	Spottail shiner	undetectable		Deepwater Sculpin	Undetectable
	Smallmouth bass	undetectable		Lake Whitefish	Undetectable
	Brook Trout	undetectable		Yellow Perch	Undetectable
**Brook Trout**	Brook Trout	23.44 ± 0.11	**Yellow Perch**	Yellow Perch	23.44 ± 0.039
	Spottail shiner	undetectable		Deepwater Sculpin	Undetectable
	Round whitefish	undetectable		Rainbow Smelt	Undetectable
	Smallmouth bass	undetectable		Lake Whitefish	Undetectable

Table demonstrates C_q_ values for qPCR experiments performed using 100 ng of DNA for each species listed. Data represented as mean C_q_ value ± standard error of mean.

## Discussion

Species-specific qPCR primer-probe sets were successfully developed and validated for the eight species of interest: lake whitefish (*Coregonus clupeaformis*), yellow perch (*Perca flavescens*), rainbow smelt (*Osmerus mordax*), brook trout (*Salvelinus fontinalis*), smallmouth bass (*Micropterus dolomieu*), round whitefish (*Prosopium cylindraceum*), spottail shiner (*Notropis hudsonius*) and deepwater sculpin (*Myoxocephalus thompsonii*). The design of novel forward/reverse primers in conjunction with probe sequences permitted for excellent species specificity. Furthermore, the selected primer-probe sets were multiplex compatible allowing for the development of rapid and high-throughput assays with uncompromised accuracy. Indeed, the development of a fully automated species-decoder algorithm allowed for target species identification with 100% accuracy while completely removing any false-positive detection of non-target species.

Fish that are entrained or impinged at once-through cooling system intake zones are often more difficult to identify because of the premature developmental stage (majority are in embryo or larval stage[2]) or because the samples are degraded, damaged and/or missing key identification traits. The developed qPCR primer-probe assays provide several advantages over traditional morphological species identification methods. Here, probe-based qPCR assays are effective at target species identification regardless of gender, life stage, cryptic variation or intraspecific phenotypic plasticity. Furthermore, Ko *et al*. 2013 illustrated that morphological based species identification methods are highly inaccurate and error-prone [4]. On the contrary, the incorporation of the automated species decoder algorithm in our probe-based qPCR analysis eliminates false identification errors due to human biases.

In addition to accuracy, large-scale industrial environmental monitoring programs require species identification assays that are cost-effective and high-throughput capable. The probe-based qPCR technique utilized in this study is substantially more cost-effective (Table 6) and time efficient (Table 7) than DNA barcoding and morphological identification methods. To identify an individual sample, qPCR was the cheapest option at $5.82 compared to an external contractor charge of $13 per sample for morphological identification and $18.79 for DNA barcoding, a difference of $7.18 and $12.97 respectively (Table 6). The cost of the primer-probe sets becomes substantially lower as the sample number increased. For example, when all wells of a 96-format qPCR machine are utilized, 96 samples can be processed for approximately $560, less than half the cost of the other methods (DNA barcoding and morphological identification methods exceed $1200). Similarly, analysis of time requirements (Table 7) reveals that qPCR is the most rapid identification technique which is capable of processing hundreds of samples in only a matter of hours. Here, preparation of the samples required approximately 1.5 h, which was considerably less than the other two techniques (Table 7). In addition, DNA barcoding required several post-PCR processing steps (gel electrophoresis and imaging) that further increased the processing time compared to qPCR. Even with the removal of the PCR verification step with gel electrophoresis for a more high-throughput system, qPCR would still be a quicker approach to yielding results. Review of the morphological identification method demonstrated that this technique is highly variable, depending on the ichthyologist’s level of expertise, as well as other factors including species, life stage and physical composition of the sample. For example, a larval Coregonine species would require significantly more time and expertise to identify than an adult burbot (*Lota lota*). Furthermore, once-through cooling systems mostly impinge embryo or larval fish where morphological identification is less effective and requires highly specialized taxonomists [2]. Taken together, Tables 6 and 7 reveals that the probe-based qPCR assay developed in this study is the most cost-effective and high throughput species identification method.

**Table 6:** Approximate cost analysis for each species identification technique. Per 1 Sample Per 96 Samples.

**Real-Time PCR Assays**		
DNA Extraction Kit	$3.39	$325.44
Primers	$0.0003	$0.03
Probes	$0.02	$1.92
Hydrolysis Mix	$1.40	$134.4
Microamp 96-well Plates	$0.01	$1.30
Labour	$1	$100
**Total**	**$5.82**	**$563.09**
**DNA Barcoding**		
DNA Extraction Kit	$3.39	$325.44
Primers	$0.0009	$0.08
iQ MasterMix	$2.81	$269.76
Agrose gel and Loading Dye	$3.04	$40.07
PCR Purfication Kit	$2.61	$250.75
Strip Tubes	$0.47	$5.64
Sanger Sequencing	$3.50	$336
Labour	$1	$100
**Total**	**$18.79**	**$1,319**
**Morphological Identification**		
Labour	$13 ($10 US)	$1,248 ($960 US)
**Total**	**$13** **($10 US)**	**$1,248** **($960 US)**

Figures are represented in CAD dollars.

**Table 7:** Approximate time requirement for each species identification technique.

	**Per 96 Samples**
**Real-Time PCR Assays**	
DNA Extraction	Prep: 1 hour
	Incubation: 3–4 hours
Real-Time PCR	Prep: 1 hour
	Run Time: 30 minutes
Analysis	30 minutes
**Total**	**6–7 hours**
**DNA Barcoding**	
DNA Extraction	Prep: 1 hour
	Incubation: 3–4 hours
PCR	Prep: 30 minutes
	Run Time: 2 hours
Gel Electrophoresis	Prep: 30 minutes
	Run Time: 1–2 hours
PCR Purification	1 hour
Analysis	1 hour
**Total**	**10–12 hours**
**Morphological Identification**	
Labour	8–16 hours
**Total**	**8–16 hours**

Morphological identification time varies depending on species complexity, species such as *Coregonus* required significantly more time and experience to identify than a burbot.

Many fish species within the Great Lakes are post-glacial and recently diverged. For example, the *Coregonus* members demonstrates recent evolutionary divergence and exhibits extensive interspecies COI haplotype sharing [7,27–29]. This genus is extremely difficult to identify morphologically as individuals from the same species exhibit multiple different morphologies within the same lake [29,30]. As well, DNA barcoding has had several limitations with identifying individuals from Coregonine to the genus and/or species level due to decreased mitochondrial DNA variation between species [7,27]. Furthermore, the *Coregonus* genus within Canada demonstrated the greatest percent homology to one another out of all the species of interest, with a 97–99% similarity (Table 1). As a result, Lake Whitefish primer-probe set had the highest sequence homology (i.e. lowest number of bp differences) between its closely related CON species (S3 Table). Overdyk *et al*. 2016 previously designed a qPCR assay to distinguish Lake Whitefish from the other *Coregonus* species; however, their assay only had one bp difference on their probe sequence for all the *Coregonus* species and had reported non-target C_q_ detection of 29 for yellow perch. Our assay had 3 to 5bp differences that were spread over the reverse primer and probe sequences and no C_q_ detection for non-target species. This demonstrates that our assay design criteria was very effective and was able to identify primer-probe sets for even difficult to discriminate species such as the lake whitefish.

The development and validation of the probe-based qPCR assay outlined in this study is translatable to all species. For example, other ecologically and economically important fish species that may be of concern for industrial environmental programs include walleye, lake trout, cisco and round goby [2]. As well, the high sensitivity achieved by the qPCR assays demonstrate that the primer-probe sets can be successfully applied to samples of low abundance, including environmental DNA (eDNA) or digested stomach samples. eDNA is novel non-invasive technique used to identify organisms by the fragments of DNA that are released in the environment. Currently, highly expensive next-generation sequencing platform has been used to detect species from low abundant eDNA samples. Our results suggest that the qPCR primer-probe sets developed in this study can potentially offer a more time efficient and cost-effective means to identify specific species from eDNA samples.

## Conclusion

Highly accurate and cost-effective species-specific qPCR hydrolysis primer-probe sets were successfully developed for the rapid and high-throughput identification of eight ecologically and economically important freshwater fish species. The combination of the species-specific primer/probes sets with an automated species decoder algorithm resulted in target species identification with 100% accuracy coupled with complete absence of false-positive detection from non-target controls. Most importantly, the probe-based qPCR assays were highly sensitive with detection limits as low as 1 picogram of sample DNA. Furthermore, the probe-based qPCR technique utilized in this study is substantially more cost-effective and time efficient than DNA barcoding and morphological identification methods. In summary, probe-based multiplex qPCR assays provide a rapid and accurate method for freshwater fish species identification, and the methodology established in this study can be utilized for various other species identification initiatives.

## Supporting information

S1 Table List of sample source, tissue-type, and number of individuals per group. (DOCX)

S2 Table Comparison of primer design methodologies using SYBR green based qPCR analysis. 100 ng of each species of interest was assayed using forward and reverse primers designed using either Method #1 or #2, and resulting C_q_ values are presented. Therefore, primer sequences for *Perca flavescens* and *Salvelinus fontinalis* were obtained from Method #2, while Method #1 was utilized the remaining six species
(DOCX)

S3 Table Sequent alignment comparison of the primer/probe sets with the corresponding species of interest, control species (same genus) and non-target species (different genus). The number of base pair mismatches are highlighted in red and also tabulated.
(DOCX)

S4 Table qPCR C_q_ values for single-plex and multiplex analysis. Each species of interest was assayed using single-plex and multiplex conditions, while the control species from the same genus were assayed using multiplex conditions only. Probe set one consisted of primer/probe sets for following species of interest: smallmouth bass, spottail shiner, round whitefish, and brook trout (S4.1 Table). Probe set two consisted of primer/probe sets for following species of interest: lake whitefish, deepwater sculpin, rainbow smelt and yellow perch (S4.2 Table). Species that were undetectable are marked as “-”.
(DOCX)
