# Supplementary material for: Development and validation of probe-based multiplex real-time PCR assays for the rapid and accurate detection of freshwater fish species
Source: PLoS One. 2019 Jan 30;14(1):e0210165. doi: 10.1371/journal.pone.0210165 (PMC6353101; doi:10.1371/journal.pone.0210165)
Supplement: S1 Table — (DOCX) [file pone.0210165.s001.docx]

**S1 Table. List of sample source, tissue-type, and number of individuals per group.**

| **Species** | **# of Samples** | **Sample Type** | **Received From** |
| --- | --- | --- | --- |
| Smallmouth Bass | 10 | fin clip | Dr. Robert Humston, Washington and Lee University |
|  | 5 | Muscle | Dr. Louis Bernatchez, Universite Laval |
| Largemouth Bass | 6 | Muscle | Dr. Nancy Denslow, University of Florida |
| Spottail Shiner | 13 | Muscle | Dr. James Johnson, USGS |
| Spottail Shiner CONs | 14 | Muscle | Dr. James Johnson, USGS |
| Round Whitefish | 6 | Muscle | Dr. Joanna Wilson, McMaster University |
|  | 5 | Liver | Dr. James Johnson, USGS |
| Pygmy Whitefish | 3 | Muscle | Dr. Hernan Lopez-Fernandez, Royal Ontario Museum |
| Brook Trout | 4 | Muscle | Dr. Louis Bernatchez, Universite Laval |
| Brook Trout CONs | 8 | Muscle | Dr. Eric B. (Rick) Taylor, University of British Columbia |
| Lake Whitefish | 5 | Muscle | Dr. Joanna Wilson, McMaster University |
| Lake Whitefish CONs | 5 | Muscle | Dr. Hernan Lopez-Fernandez, Royal Ontario Museum |
| Deepwater Sculpin | 5 | Muscle | Dr. Hernan Lopez-Fernandez, Royal Ontario Museum |
| Fourhorn Sculpin | 2 | Muscle | Dr. Hernan Lopez-Fernandez, Royal Ontario Museum |
| Rainbow Smelt | 14 | Muscle | Dr. Doug Watkinson, Department of Fisheries and Oceans |
| Yellow Perch | 11 | muscle | Andrew Zarnke, Laurentian University |
| Yellow Perch CONs | 7 | muscle | Dr. Hernan Lopez-Fernandez, Royal Ontario Museum |
