# Supplementary material for: Development and validation of probe-based multiplex real-time PCR assays for the rapid and accurate detection of freshwater fish species
Source: PLoS One. 2019 Jan 30;14(1):e0210165. doi: 10.1371/journal.pone.0210165 (PMC6353101; doi:10.1371/journal.pone.0210165)
Supplement: S2 Table — 100 ng of each species of interest was assayed using forward and reverse primers designed using either Method #1 or #2, and resulting Cq values are presented. Therefore, primer sequences for Perca flavescens and Salvelinus fontinalis were obtained from Method #2, while Method #1 was utilized the remaining six species (DOCX) [file pone.0210165.s002.docx]

**S2 Table**. **Comparison of primer design methodologies using SYBR green based qPCR analysis.**

| **Species** | **Method #1** | **Method #2** |
| --- | --- | --- |
| ***Coregonus clupeaformis*** | 16.17 | 17.24 |
| ***Micropterus dolomieu*** | 14.98 | 15.13 |
| ***Myoxocephalus thompsonii*** | 15.62 | 18.86 |
| ***Notropis hudsonius*** | 12.72 | 13.08 |
| ***Osmerus mordax*** | 18.36 | 17.83 |
| ***Perca flavescens*** | 17.89 | 18.98 |
| ***Prosopium cylindraceum*** | 15.03 | 15.62 |
| ***Salvelinus fontinalis*** | 16.33 | 15.40 |

100 ng of each species of interest was assayed using forward and reverse primers designed using either Method #1 or #2, and resulting C_q_ values are presented. Therefore, primer sequences for *Perca flavescens* and *Salvelinus fontinalis* were obtained from Method #2, while Method #1 was utilized the remaining six species
