# Supplementary material for: Development and validation of probe-based multiplex real-time PCR assays for the rapid and accurate detection of freshwater fish species
Source: PLoS One. 2019 Jan 30;14(1):e0210165. doi: 10.1371/journal.pone.0210165 (PMC6353101; doi:10.1371/journal.pone.0210165)
Supplement: S3 Table — The number of base pair mismatches are highlighted in red and also tabulated. (DOCX) [file pone.0210165.s003.docx]

**S3 Table**. **Sequent alignment comparison of the primer/probe sets with the corresponding species of interest, control species (same genus) and non-target species (different genus).** The number of base pair mismatches are highlighted in red and also tabulated.

|  | **Smallmouth Bass (Micopterus dolomieu) Primer-Probe Set** | | | | | |
| --- | --- | --- | --- | --- | --- | --- |
|  | **Forward Primer** | | **Reverse Compl. Primer** | | **Internal Oligo** | |
|  | **TCTTTCCTTCTCCTGCTCGC** | | **TTCATCTTGCGGGTGTCTCC** | | **GCTGGAGCTGGCACTGGGTG** | |
|  | Aligned Primer | # of mismatches | Aligned Primer | # of mismatches | Aligned Primer | # of mismatches |
| ***Micropterus dolomieu*** | TCTTTCCTTCTCCTGCTCGC | 0 | TTCATCTTGCGGGTGTCTCC | 0 | GCTGGAGCTGGCACTGGGTG | 0 |
| ***Coregonus clupeaformis*** | TCCTTTCTCCTTCTCCTGGC | 6 | TCCACTTAGCTGGTATTTCC | 7 | GCCGGTGCCGGCACAGGATG | 5 |
| ***Myoxocephalus thompsonii*** | TCCTTTCTACTTCTCTTAGC | 7 | TACATCTAGCAGGAATCTCT | 6 | GCAGGGGCAGGAACCGGGTG | 5 |
| ***Notropis hudsonius*** | TCATTTCTACTATTATTAGC | 8 | TTCACCTAGCAGGTGTCTCA | 4 | GCTGGGGCTGGAACAGGATG | 4 |
| ***Osmerus mordax*** | TCCTTTCTTCTTCTCTTAGC | 6 | TTCACCTTGCGGGGATCTCC | 2 | GCAGGCGCCGGGACTGGTTG | 5 |
| ***Perca flavescens*** | TCTTTCCTTCTCCTCCTTGC | 2 | TACACCTTGCGGGGATCTCC | 4 | GCCGGAGCTGGTACCGGATG | 4 |
| ***Prosopium cylindraceum*** | TCCTTTCTTCTTCTCCTGGC | 5 | TACACTTAGCTGGTATTTCC | 7 | GCCGGCGCCGGCACAGGATG | 5 |
| ***Salvelinus fontinalis*** | TCCTTTCTACTTCTCCTGGC | 6 | TACATTTAGCTGGCATTTCC | 7 | GCCGGCGCCGGTACGGGGTG | 5 |
| **Largemouth Bass**  **(*M. salmoides)*** | TCCTTCCTTCTCCTGCTCGC | 1 | TTCACCTTGCTGGTGTCTCC | 2 | GCCGGGGCTGGCACTGGGTG | 2 |

|  | **Spottail shiner (Notropis hudsonius) Primer/Probe Set** | | | | | |
| --- | --- | --- | --- | --- | --- | --- |
|  | **Forward Primer** | | **Reverse Compl. Primer** | | **Internal Oligo** | |
|  | **CTATTATTAGCTTCTTCTGGGGTTG** | | **GGGCGCATCAGTAGACCTC** | | **GCAGGCAATCTTGCCCACGC** | |
|  | Aligned Primer | # of mismatches | Aligned Primer | # of mismatches | Aligned Primer | # of mismatches |
| ***Notropis hudsonius*** | CTATTATTAGCTTCTTCTGGGGTTG | 0 | GGGCGCATCAGTAGACCTC | 0 | GCAGGCAATCTTGCCCACGC | 0 |
| ***Coregonus clupeaformis*** | CTTCTCCTGGCCTCGTCCGGAGTTG | 9 | AGGAGCCTCCGTCGATTTA | 8 | GCAGGCAACCTCGCCCACGC | 2 |
| ***Micropterus dolomieu*** | CTCCTGCTCGCCTCTTCCGGGGTCG | 8 | AGGAGCATCCGTTGACCTA | 5 | GCCGGCAACCTGGCCCATGC | 4 |
| ***Myoxocephalus thompsonii*** | CTTCTCTTAGCCTCTTCGGGGGTTG | 5 | GGGAGCCTCTGTTGACCTA | 5 | GCCGGAAACCTGGCCCACGC | 4 |
| ***Osmerus mordax*** | CTTCTCTTAGCTTCTTCCGGGGTTG | 4 | GGGAGCTTCCGTAGATTTA | 6 | GCTGGCAATTTGGCCCACGC | 3 |
| ***Perca flavescens*** | CTCCTCCTTGCTTCCTCAGGAGTTG | 8 | TGGAGCATCTGTTGATTTA | 7 | GCTGGGAACTTAGCACATGC | 7 |
| ***Prosopium cylindraceum*** | CTTCTCCTGGCCTCATCCGGAGTTG | 9 | AGGGGCCTCCGTTGACTTA | 6 | GCAGGCAACCTCGCTCACGC | 3 |
| ***Salvelinus fontinalis*** | CTTCTCCTGGCTTCGTCCGGAGTTG | 8 | AGGAGCTTCCGTTGATTTA | 8 | GCTGGGAACCTCGCCCACGC | 4 |
| **Pugnose Shiner**  **(*N. anogenus*)** | CTATTATTAGCCTCTTCTGGCGTTG | 2 | AGGAGCATCAGTAGACCTC | 2 | GCGGGTAATCTTGCCCATGC | 3 |
| **Emerald Shiner**  **(*N. atherinoides*)** | TTACTATTAGCCTCCTCTGGTGTTG | 5 | AGGAGCGTCAGTAGACCTA | 4 | TCAGGAAACCTTGCCCACGC | 3 |
| **Bridle Shiner**  **(*N. bifrenatus*)** | TTACTATTAGCCTCTTCTGGAGTTG | 4 | AGGAGCATCAGTAGATCTT | 4 | GCAGGTAACCTTGCCCATGC | 3 |
| **River Shiner**  **(*N. blennius*)** | TTATTACTAGCCTCTTCTGGAGTTG | 4 | AGGCGCATCAGTAGACCTT | 2 | GCAGGCAACCTTGCCCACGC | 1 |
| **Ghost Shiner**  **(*N. buchanani*)** | CTACTACTAGCCTCTTCTGGTGTTG | 4 | AGGAGCGTCAGTAGACCTA | 4 | GCGGGTAACCTTGCCCACGC | 3 |
| **Bigmouth Shiner**  **(*N. dorsalis*)** | TTATTACTAGCTTCTTCAGGTGTCG | 5 | AGGAGCGTCAGTCGACCTC | 4 | GCAGGCAATCTCGCTCACGC | 2 |
| **Blackchin Shiner**  **(*N. heterodon*)** | TTACTCCTAGCCTCTTCTGGTGTTG | 6 | AGGAGCATCAGTAGACCTT | 3 | GCAGGTAATCTTGCCCATGC | 2 |
| **Blacknose Shiner**  **(*N. heterolepis*)** | TTACTATTAGCCTCTTCTGGTGTTG | 4 | AGGAGCATCAGTAGACCTC | 2 | GCAGGTAACCTTGCCCACGC | 2 |
| **Carmine Shiner**  **(*N. percobromus*)** | CTACTACTAGCCTCTTCCGGTGTTG | 5 | AGGAGCATCAGTAGACCTA | 3 | TCAGGGAACCTTGCCCACGC | 3 |
| **Silver Shiner**  **(*N. photogenis*)** | TTACTATTAGCCTCTTCTGGCGTTG | 4 | AGGAGCATCAGTAGACCTC | 2 | GCGGGTAATCTTGCCCACGC | 2 |
| **Rosyface Shiner**  **(*N. rubellus*)** | TTATTACTAGCCTCTTCTGGTGTTG | 4 | AGGAGCATCAGTAGACCTA | 3 | TCAGGAAACCTTGCCCATGC | 4 |
| **Sand Shiner**  **(*N. stramineus*)** | CTACTGCTAGCCTCTTCTGGTGTCG | 6 | AGGGGCATCTGTAGATCTC | 4 | GCAGGCAATCTTGCCCACGC | 0 |
| **Weed Shiner**  **(*N. texanus*)** | CTACTGCTAGCCTCTTCTGGTGTTG | 5 | AGGGGCATCAGTAGACCTT | 3 | GCAGGTAACCTCGCTCACGC | 4 |
| **Mimic Shiner**  **(*N. volucellus*)** | CTACTACTAGCCTCTTCTGGTGTTG | 4 | AGGAGCGTCAGTAGACCTA | 4 | GCGGGTAACCTTGCCCACGC | 3 |

|  | Round Whitefish (*Prosopium cylindraceum*) Primer/Probe Set | | | | | |
| --- | --- | --- | --- | --- | --- | --- |
|  | **Forward Primer** | | **Reverse Compl.Primer** | | **Internal Oligo** | |
|  | **AATGTAATCGTCACGGCCCA** | | **CCCGATATAGCATTCCCCCG** | | **TGACTAATTCCCCTTATGATCGGAGCA** | |
|  | Aligned Primer | # of mismatches | Aligned Primer | # of mismatches | Aligned Primer | # of mismatches |
| ***Prosopium cylindraceum*** | AATGTAATCGTCACGGCCCA | 0 | CCCGATATAGCATTCCCCCG | 0 | TGACTAATTCCCCTTATGATCGGAGCA | 0 |
| ***Coregonus clupeaformis*** | AATGTGATCGTCACGGCCCA | 1 | CCCGACATGGCATTTCCCCG | 3 | TGATTAATCCCACTTATAATCGGGGCC | 6 |
| ***Micropterus dolomieu*** | AATGTAATTGTTACAGCGCA | 4 | CCCGACATAGCATTCCCTCG | 2 | TGACTTATCCCCCTAATGATCGGTGCC | 5 |
| ***Myoxocephalus thompsonii*** | AACGTAATTGTTACAGCTCA | 5 | CCTGACATGGCTTTTCCCCG | 5 | TGACTCATCCCCTTAATGATTGGGGCC | 6 |
| ***Notropis hudsonius*** | AACGTTATCGTTACTGCCCA | 4 | CCTGATATAGCATTTCCACG | 3 | TGACTTGTACCTCTAATGATCGGAGCA | 5 |
| ***Osmerus mordax*** | AATGTTATCGTCACCGCGCA | 3 | CCAGATATGGCCTTCCCTCG | 4 | TGGCTCATCCCCCTTATGATTGGGGCC | 6 |
| ***Perca flavescens*** | AACGTAATTGTTACAGCACA | 5 | CCTGACATAGCTTTCCCTCG | 4 | TGACTAATTCCACTTATGATCGGTGCC | 3 |
| ***Salvelinus fontinalis*** | AACGTAATCGTAACAGCCCA | 3 | CCAGACATAGCATTCCCTCG | 3 | TGATTAATTCCTCTAATAATTGGAGCC | 6 |
| **Pygmy Whitefish**  **(*P. coulterii*)** | AATGTGATCGTTACAGCCCA | 3 | CCCGATATAGCATTTCCCCG | 1 | TGATTAATCCCCCTTATGATTGGGGCA | 4 |
| **Mountain Whitefish**  **(*P. williamsoni*)** | AATGTAATCGTTACAGCCCA | 2 | CCCGATATAGCATTTCCCCG | 1 | TGACTAATCCCCCTTATGATCGGAGCA | 1 |

|  | **Brook Trout (Salvelinus fontinalis) Primer/Probe Set** | | | | | |
| --- | --- | --- | --- | --- | --- | --- |
|  | **Forward Primer** | | **Reverse Compl.Primer** | | **Internal Oligo** | |
|  | **CGGTACGGGGTGAACAGTTT** | | **CCCTACATTTAGCTGGCATTTCC** | | **CTCGCCCACGCAGGAGCTTC** | |
|  | Aligned Primer | # of mismatches | Aligned Primer | # of mismatches | Aligned Primer | # of mismatches |
| ***Salvelinus fontinalis*** | CGGTACGGGGTGAACAGTTT | 0 | CCCTACATTTAGCTGGCATTTCC | 0 | CTCGCCCACGCAGGAGCTTC | 0 |
| ***Coregonus clupeaformis*** | CGGCACAGGATGAACAGTCT | 4 | CCCTCCACTTAGCTGGTATTTCC | 3 | CTCGCCCACGCAGGAGCCTC | 1 |
| ***Micropterus dolomieu*** | TGGCACTGGGTGAACTGTCT | 5 | CTCTTCATCTTGCGGGTGTCTCC | 8 | CTGGCCCATGCAGGAGCATC | 3 |
| ***Myoxocephalus thompsonii*** | AGGAACCGGGTGGACAGTAT | 5 | CCTTACATCTAGCAGGAATCTCT | 6 | CTGGCCCACGCGGGAGCCTC | 3 |
| ***Notropis hudsonius*** | TGGAACAGGATGAACTGTCT | 6 | CTCTTCACCTAGCAGGTGTCTCA | 9 | CTTGCCCACGCGGGCGCATC | 4 |
| ***Osmerus mordax*** | CGGGACTGGTTGAACAGTCT | 4 | CTCTTCACCTTGCGGGGATCTCC | 8 | TTGGCCCACGCGGGAGCTTC | 3 |
| ***Perca flavescens*** | TGGTACCGGATGAACTGTTT | 4 | CTTTACACTTAGCAGGGGTTTCC | 6 | TTAGCACATGCTGGAGCATC | 6 |
| ***Prosopium cylindraceum*** | CGGCACAGGATGAACAGTGT | 4 | CCCTACACTTAGCTGGTATTTCC | 2 | CTCGCTCACGCAGGGGCCTC | 3 |
| **Arctic Char**  **(*S. alpinus*)** | CGGTACGGGATGGACAGTCT | 3 | CCCTTCATTTAGCTGGCATTTCC | 1 | CTCGCCCACGCAGGGGCCTC | 2 |
| **Bull Trout**  **(*S.confluentus*)** | CGGTACGGGATGGACAGTCT | 3 | CCCTTCATTTAGCTGGCATTTCC | 1 | CTCGCCCACGCAGGGGCCTC | 2 |
| **Dolly Varden**  **(*S. malma*)** | CGGTACGGGATGGACAGTCT | 3 | CCCTTCATTTAGCTGGCATTTCC | 1 | CTCGCCCACGCAGGAGCCTC | 1 |
| **Lake Trout**  **(*S. namaycush*)** | CGGTACGGGATGAACAGTCT | 2 | CTCTTCATTTAGCTGGCATTTCC | 2 | CTCGCCCACGCAGGGGCCTC | 2 |

|  | **Lake Whitefish (Coregonus clupeaformis) Primer/Probe Set** | | | | | |
| --- | --- | --- | --- | --- | --- | --- |
|  | **Forward Primer** | | **Reverse Compl.Primer** | | **Internal Oligo** | |
|  | **TCTCCCTCCACTTAGCTGGT** | | **ACCCCTCTTTTTGTCTGGGC** | | **TTCCTCTATCTTGGGGGCCGTT** | |
|  | Aligned Primer | # of mismatches | Aligned Primer | # of mismatches | Aligned Primer | # of mismatches |
| ***Coregonus clupeaformis*** | TCTCCCTCCACTTAGCTGGT | 0 | ACCCCTCTTTTTGTCTGGGC | 0 | TTCCTCTATCTTGGGGGCCGTT | 0 |
| ***Micropterus dolomieu*** | TCTCTCTTCATCTTGCGGGT | 6 | ACACCCCTGTTTGTTTGGTC | 5 | CTCCTCCATCCTAGGGGCCATC | 6 |
| ***Myoxocephalus thompsonii*** | TCTCCTTACATCTAGCAGGA | 5 | ACCCCTCTATTCGTGTGATC | 5 | CTCTTCGATCCTCGGAGCAATC | 9 |
| ***Notropis hudsonius*** | TCTCTCTTCACCTAGCAGGT | 4 | ACACCTCTTTTCGTATGAGC | 4 | CTCATCAATTCTAGGGGCAGTT | 7 |
| ***Osmerus mordax*** | TCTCTCTTCACCTTGCGGGG | 6 | ACCCCCTTATTTGTCTGAGC | 4 | CTCCTCTATTCTCGGGGCAATT | 6 |
| ***Perca flavescens*** | TCTCTTTACACTTAGCAGGG | 5 | ACTCCCTTGTTCGTATGGGC | 5 | TTCCTCAATTCTAGGTGCTATT | 7 |
| ***Prosopium cylindraceum*** | TCTCCCTACACTTAGCTGGT | 1 | ACACCCCTTTTTGTTTGAGC | 4 | TTCCTCTATTTTAGGAGCCGTT | 3 |
| ***Salvelinus fontinalis*** | TTTCCCTACATTTAGCTGGC | 4 | ACCCCACTTTTTGTTTGAGC | 3 | TTCCTCAATTTTAGGAGCCATT | 5 |
| **Cisco (*C. artedi*)** | TCTCCCTCCACTTAGCTGGT | 0 | ACCCCTCTTTTTGTCTGAGC | 1 | TTCCTCTATCTTAGGAGCCGTT | 2 |
| **Arctic Cisco**  **(*C. autumnalis*)** | TCTCCCTCCACTTAGCTGGT | 0 | ACCCCTCTTTTTGTCTGAGC | 1 | TTCCTCTATCTTAGGAGCCGTT | 2 |
| **Bloater**  **(*C. hoyi*)** | TCTCCCTCCACTTAGCTGGT | 0 | ACCCCCCTGTTTGTCTGAGC | 3 | TTCCTCTATCTTAGGAGCCGTT | 2 |
| **Atlantic Whitefish**  **(*C. huntsmani*)** | TCTCCCTCCACCTAGCTGGT | 1 | ACCCCTCTTTTTGTTTGAGC | 2 | TTCCTCTATCTTGGGAGCCGTT | 1 |
| **Kiyi (*C. kiyi*)** | TCTCCCTCCACTTAGCTGGT | 0 | ACCCCTCTTTTTGTCTGAG | 1 | TTCCTCTATCTTAGGAGCCGTT | 2 |
| **Bering Cisco**  **(*C. laurettae*)** | TCTCCCTCCACTTAGCTGGT | 0 | ACCCCTCTTTTTGTCTGAGC | 1 | TTCCTCTATCTTAGGAGCCGTT | 2 |
| **Broad Whitefish**  **(*C. nasus*)** | TCTCCCTCCACTTAGCTGGT | 0 | ACCCCTCTTTTTGTCTGGGC | 0 | TTCCTCTATCTTAGGGGCCGTT | 1 |
| **Blackfin Cisco**  **(*C. nigripinnis*)** | TCTCCCTCCACTTAGCTGGT | 0 | ACCCCTCTTTTTGTCTGAGC | 1 | TTCCTCTATCTTAGGAGCCGTT | 2 |
| **Sardine Cisco**  **(*C. sardinella*)** | TCTCCCTCCACTTAGCTGGT | 0 | ACCCCTCTTTTTGTCTGAGC | 1 | TTCCTCTATCTTAGGGGCCGTT | 1 |
| **Shortjaw Cisco**  **(*C. zenithicus*)** | TCTCCCTCCACTTAGCTGGT | 0 | ACCCCTCTTTTTGTCTGAGC | 1 | TTCCTCTATCTTAGGAGCCGTT | 2 |

|  | Deepwater Sculpin (*Myoxocephalus thompsonii*) Primer/Probe Set | | | | | |
| --- | --- | --- | --- | --- | --- | --- |
|  | **Forward Primer** | | **Reverse Compl. Primer** | | **Internal Oligo** | |
|  | **CTTAGCCTCTTCGGGGGTTG** | | **TCTCTTCGATCCTCGGAGCA** | | **CCACGCGGGAGCCTCTGTTG** | |
|  | Aligned Primer | # of mismatches | Aligned Primer | # of mismatches | Aligned Primer | # of mismatches |
| ***Myoxocephalus thompsonii*** | CTTAGCCTCTTCGGGGGTTG | 0 | TCTCTTCGATCCTCGGAGCA | 0 | CCACGCGGGAGCCTCTGTTG | 0 |
| ***Coregonus clupeaformis*** | CCTGGCCTCGTCCGGAGTTG | 5 | TTTCCTCTATCTTGGGGGCC | 7 | CCACGCAGGAGCCTCCGTCG | 3 |
| ***Micropterus dolomieu*** | GCTCGCCTCTTCCGGGGTCG | 5 | TCTCCTCCATCCTAGGGGCC | 5 | CCATGCAGGAGCATCCGTTG | 4 |
| ***Notropis hudsonius*** | ATTAGCTTCTTCTGGGGTTG | 3 | TCTCATCAATTCTAGGGGCA | 5 | CCACGCGGGCGCATCAGTAG | 4 |
| ***Osmerus mordax*** | CTTAGCTTCTTCCGGGGTTG | 2 | TCTCCTCTATTCTCGGGGCA | 4 | CCACGCGGGAGCTTCCGTAG | 3 |
| ***Perca flavescens*** | CCTTGCTTCCTCAGGAGTTG | 6 | TTTCCTCAATTCTAGGTGCT | 7 | ACATGCTGGAGCATCTGTTG | 4 |
| ***Prosopium cylindraceum*** | CCTGGCCTCATCCGGAGTTG | 5 | TTTCCTCTATTTTAGGAGCC | 7 | TCACGCAGGGGCCTCCGTTG | 4 |
| ***Salvelinus fontinalis*** | CCTGGCTTCGTCCGGAGTTG | 6 | TTTCCTCAATTTTAGGAGCC | 7 | CCACGCAGGAGCTTCCGTTG | 3 |
| **Fourhorn Sculpin (*M. quadricornis*)** | CTTAGCCTCTTCGGGGGTTG | 0 | TCTCTTCAATCCTCGGAGCA | 1 | CCACGCGGGAGCCTCTGTTG | 0 |

|  | **Rainbow Smelt (Osmerus mordax) Primer/Probe Set** | | | | | |
| --- | --- | --- | --- | --- | --- | --- |
|  | **Forward Primer** | | **Reverse Compl. Primer** | | **Internal Oligo** | |
|  | **CGATTATGATCGGCGGGTTTG** | | **GATATGGCCTTCCCTCGCAT** | | **CCCCCTTATGATTGGGGCCCCA** | |
|  | Aligned Primer | # of mismatches | Aligned Primer | # of mismatches | Aligned Primer | # of mismatches |
| ***Osmerus mordax*** | CGATTATGATCGGCGGGTTTG | 0 | GATATGGCCTTCCCTCGCAT | 0 | CCCCCTTATGATTGGGGCCCCA | 0 |
| ***Coregonus clupeaformis*** | CAATTATGATTGGAGGCTTTG | 4 | GACATGGCATTTCCCCGAAT | 5 | CCCACTTATAATCGGGGCCCCC | 4 |
| ***Micropterus dolomieu*** | CCATTATAATTGGAGGCTTTG | 5 | GACATAGCATTCCCTCGAAT | 4 | CCCCCTAATGATCGGTGCCCCC | 4 |
| ***Myoxocephalus thompsonii*** | CAATCATAATTGGGGGTTTCG | 8 | GACATGGCTTTTCCCCGAAT | 5 | CCCCTTAATGATTGGGGCCCCT | 3 |
| ***Notropis hudsonius*** | CAATTCTTATTGGCGGATTTG | 5 | GATATAGCATTTCCACGAAT | 5 | ACCTCTAATGATCGGAGCACCT | 7 |
| ***Perca flavescens*** | CAATTATGATTGGGGGCTTTG | 4 | GACATAGCTTTCCCTCGAAT | 4 | TCCACTTATGATCGGTGCCCCT | 5 |
| ***Prosopium cylindraceum*** | CAATTATGATTGGAGGATTTG | 4 | GATATAGCATTCCCCCGAAT | 4 | TCCCCTTATGATCGGAGCACCC | 5 |
| ***Salvelinus fontinalis*** | CAATTATGATTGGAGGATTTG | 4 | GACATAGCATTCCCTCGAAT | 4 | TCCTCTAATAATTGGAGCCCCA | 5 |
| **Pacific Rainbow**  **Smelt (*O. dentex*)** | CAATCATGATTGGAGGTTTCG | 6 | GACATGGCCTTCCCCCGTAT | 3 | CCCCCTTATGATTGGGGCCCCA | 0 |

|  | **Yellow Perch (Perca flavescens) Primer/Probe Set** | | | | | |
| --- | --- | --- | --- | --- | --- | --- |
|  | **Forward Primer** | | **Reverse Compl.Primer** | | **Internal Oligo** | |
|  | **GATCGGTGCCCCTGACATAG** | | **TTATCCCCCTCTTGCTGGGA** | | **AAGCCGGAGCTGGTACCGGA** | |
|  | Aligned Primer | # of mismatches | Aligned Primer | # of mismatches | Aligned Primer | # of mismatches |
| ***Perca flavescens*** | GATCGGTGCCCCTGACATAG | 0 | TTATCCCCCTCTTGCTGGG | 0 | AAGCCGGAGCTGGTACCGGA | 0 |
| ***Coregonus clupeaformis*** | AATCGGGGCCCCCGACATGG | 4 | CTACCCCCCTCTGGCAGGC | 5 | AAGCCGGTGCCGGCACAGGA | 4 |
| ***Micropterus dolomieu*** | GATCGGTGCCCCCGACATAG | 1 | CTACCCCCCTCTTGCCGGC | 4 | AAGCTGGAGCTGGCACTGGG | 4 |
| ***Myoxocephalus thompsonii*** | GATTGGGGCCCCTGACATGG | 3 | ATACCCTCCCCTTGCCGGA | 6 | AAGCAGGGGCAGGAACCGGG | 5 |
| ***Notropis hudsonius*** | GATCGGAGCACCTGATATAG | 3 | CTACCCCCCACTTGCAGGC | 5 | AAGCTGGGGCTGGAACAGGA | 4 |
| ***Osmerus mordax*** | GATTGGGGCCCCAGATATGG | 5 | CTATCCCCCACTTGCTGGC | 3 | AAGCAGGCGCCGGGACTGGT | 6 |
| ***Prosopium cylindraceum*** | GATCGGAGCACCCGATATAG | 4 | GTATCCCCCACTAGCAGGC | 5 | AAGCCGGCGCCGGCACAGGA | 4 |
| ***Salvelinus fontinalis*** | AATTGGAGCCCCAGACATAG | 4 | TTACCCCCCTCTAGCTGGG | 2 | AAGCCGGCGCCGGTACGGGG | 4 |
| **Logperch**  **(*Percina caprodes*)** | GATCGGCGCCCCCGATATGG | 4 | CTACCCGCCTTTAGCGGGA | 7 | AAGCAGGGGCTGGAACTGGG | 5 |
| **Channel Darter (*Percina copelandi*)** | GATCGGCGCCCCCGACATGG | 3 | ATACCCACCTCTGGCTGGG | 4 | AGGCTGGAGCTGGAACCGGA | 3 |
| **Blackside Darter (*Percina maculata*)** | GATTGGTGCCCCCGACATGG | 3 | CTACCCGCCCCTGGCTGGA | 6 | AAGCTGGGGCTGGAACCGGA | 3 |
| **River Darter**  **(*Percina shumardi*)** | GATCGGTGCCCCCGACATGG | 2 | TTACCCGCCTCTGGCCGGA | 5 | AAGCTGGAGCTGGAACTGGA | 3 |
