# Supplementary material for: Development and validation of probe-based multiplex real-time PCR assays for the rapid and accurate detection of freshwater fish species
Source: PLoS One. 2019 Jan 30;14(1):e0210165. doi: 10.1371/journal.pone.0210165 (PMC6353101; doi:10.1371/journal.pone.0210165)
Supplement: S4 Table — Each species of interest was assayed using single-plex and multiplex conditions, while the control species from the same genus were assayed using multiplex conditions only. Probe set one consisted of primer/probe sets for following species of interest: smallmouth bass, spottail shiner, round whitefish, and brook trout (S4.1 Table). Probe set two consisted of primer/probe sets for following species of interest: lake whitefish, deepwater sculpin, rainbow smelt and yellow perch (S4.2 Table). Species that were undetectable are marked as “-”. (DOCX) [file pone.0210165.s004.docx]

**S4 Table**. **qPCR C_q_ values for single-plex and multiplex analysis.** Each species of interest was assayed using single-plex and multiplex conditions, while the control species from the same genus were assayed using multiplex conditions only. Probe set one consisted of primer/probe sets for following species of interest: smallmouth bass, spottail shiner, round whitefish, and brook trout (S4.1 Table). Probe set two consisted of primer/probe sets for following species of interest: lake whitefish, deepwater sculpin, rainbow smelt and yellow perch (S4.2 Table). Species that were undetectable are marked as “-”.

**S4.1 Table**

| **Smallmouth Bass Primer-Probe Set** | | | |  | **Spottail Shiner Primer-Probe Set** | | | | | | | |
| --- | --- | --- | --- | --- | --- | --- | --- | --- | --- | --- | --- | --- |
|  | **Smallmouth Bass** | | **Largemouth Bass** |  |  | **Spottail Shiner** | | **Bigmouth Shiner** | **Carmine Shiner** | **Rosyface Shiner** | | **Weed Shiner** |
| **[DNA] ng** | **Single Plexing** | **Multiplexing** |  |  | **[DNA] ng** | **Single Plexing** | **Multiplexing** |  |  |  |  |  |
| 300.0 | 20.87 | 20.78 | - |  | 300.0 | 16.64 | 16.73 | 38.2 | - | - | | - |
| 100.0 | 22.90 | 23.15 | - |  | 100.0 | 18.03 | 18.07 | - | - | - | | - |
| 30.00 | 25.25 | 25.71 | - |  | 30.00 | 19.70 | 19.79 | - | - | - | | - |
| 10.00 | 28.03 | 28.19 | - |  | 10.00 | 21.27 | 21.26 | - | - | - | | - |
| 3.00 | 30.67 | 30.92 | - |  | 3.00 | 23.00 | 22.99 | - | - | - | | - |
| 1.00 | 33.14 | 33.08 | - |  | 1.00 | 24.77 | 24.80 | - | - | - | | - |
| 0.30 | 36.73 | 37.28 | - |  | 0.30 | 26.72 | 26.89 | - | - | - | | - |
| 0.10 | 39.55 | 39.74 | - |  | 0.10 | 29.27 | 29.29 | - | - | - | | - |
| 0.030 | - | - | - |  | 0.030 | 31.31 | 31.47 | - | - | - | | - |
| 0.010 | - | - | - |  | 0.010 | 34.12 | 34.66 | - | - | - | | - |
| 0.003 | - | - | - |  | 0.003 | 35.85 | 35.63 | - | - | - | | - |
| 0.001 | - | - | - |  | 0.001 | 36.85 | 36.46 | - | - | - | | - |
|  |  |  |  |  |  |  |  |  |  |  | |  |
| **Round Whitefish Primer-Probe Set** | | | |  | **Brook Trout Primer-Probe Set** | | | | | | | |
|  | **Round Whitefish** | | **Pygmy Whitefish** |  |  | **Brook Trout** | | **Lake Trout** | **Arctic Char** | **Bull Trout** | **Dolly Varden** | |
| **[DNA] ng** | **Single Plexing** | **Multiplexing** |  |  | **[DNA] ng** | **Single Plexing** | **Multiplexing** |  |  |  |  |  |
| 300.0 | 19.8 | 19.9 | 35.80 |  | 300.0 | 18.53 | 18.7 | 36.4 | 36.9 | 34.7 | 38.5 | |
| 100.0 | 22.6 | 22.5 | 35.48 |  | 100.0 | 20.36 | 20.5 | 38.3 | 38.5 | 36.7 | 39.7 | |
| 30.00 | 25.2 | 25.2 | - |  | 30.00 | 22.11 | 22.3 | - | 39.9 | 38.0 | - | |
| 10.00 | 27.6 | 27.4 | - |  | 10.00 | 23.58 | 23.9 | 39.5 | - | 39.2 | - | |
| 3.00 | 30.2 | 30.1 | - |  | 3.00 | 25.38 | 25.7 | - | - | 39.7 | - | |
| 1.00 | 32.9 | 33.1 | - |  | 1.00 | 27.10 | 27.3 | - | - | - | - | |
| 0.30 | 35.3 | 35.1 | - |  | 0.30 | 28.98 | 29.2 | - | - | - | - | |
| 0.10 | 37.9 | 39.2 | - |  | 0.10 | 31.04 | 31.4 | - | - | - | - | |
| 0.030 | - | - | - |  | 0.030 | 33.09 | 33.3 | - | - | - | - | |
| 0.010 | - | - | - |  | 0.010 | 34.99 | 35.1 | - | - | - | - | |
| 0.003 | - | - | - |  | 0.003 | 37.90 | 36.6 | - | - | - | - | |
| 0.001 | - | - | - |  | 0.001 | 39.48 | 38.6 | - | - | - | - | |

**S4.2 Table**

| **Lake Whitefish Primer-Probe Set** | | | | | | | |  | **Deepwater Sculpin Primer-Probe Set** | | |
| --- | --- | --- | --- | --- | --- | --- | --- | --- | --- | --- | --- |
|  | **Lake Whitefish** | | **Cisco** | **Bloater** | | **Kiyi** | |  |  | **Deepwater Sculpin** | |
| **[DNA] ng** | **Single Plexing** | **Multiplexing** |  |  |  |  |  |  | **[DNA] ng** | **Single Plexing** | **Multiplexing** |
| 300.00 | 15.9 | 16.0 | - | - | | - | |  | 300.00 | 16.0 | 16.6 |
| 100.00 | 17.6 | 17.9 | - | - | | - | |  | 100.00 | 18.0 | 18.6 |
| 30.00 | 19.8 | 19.7 | - | - | | - | |  | 30.00 | 20.0 | 20.7 |
| 10.00 | 22.7 | 22.0 | - | - | | - | |  | 10.00 | 22.2 | 22.5 |
| 3.00 | 23.9 | 24.1 | - | - | | - | |  | 3.00 | 24.0 | 24.7 |
| 1.00 | 25.8 | 26.0 | - | - | | - | |  | 1.00 | 26.1 | 26.9 |
| 0.30 | 28.1 | 28.3 | - | - | | - | |  | 0.30 | 28.5 | 30.5 |
| 0.10 | 30.3 | 30.6 | - | - | | - | |  | 0.10 | 30.7 | 31.4 |
| 0.030 | 31.9 | 31.4 | - | - | | - | |  | 0.030 | 32.6 | 33.2 |
| 0.010 | 33.9 | 34.1 | - | - | | - | |  | 0.010 | 34.2 | 35.0 |
| 0.0030 | 35.8 | 36.1 | - | - | | - | |  | 0.0030 | 36.5 | 37.3 |
| 0.0010 | 37.5 | 37.5 | - | - | | - | |  | 0.0010 | 38.1 | 38.2 |
|  |  |  |  |  | |  | |  |  |  |  |
| **Yellow Perch Primer-Probe Set** | | | | | | | |  | **Rainbow Smelt Primer-Probe Set** | | |
|  | **Yellow Perch** | | **Log Perch** | | **Blackside Darter** | | **River Darter** |  |  | **Rainbow Smelt** | |
| **[DNA] ng** | **Single Plexing** | **Multiplexing** |  |  |  |  |  |  | **[DNA] ng** | **Single Plexing** | **Multiplexing** |
| 300.00 | 17.7 | 17.5 | 37.0 | | 35.5 | | 35.7 |  | 300.00 | 19.4 | 19.5 |
| 100.00 | 19.3 | 19.3 | - | | 37.1 | | - |  | 100.00 | 20.9 | 21.2 |
| 30.00 | 21.0 | 20.9 | - | | 36.4 | | - |  | 30.00 | 22.3 | 22.6 |
| 10.00 | 22.6 | 22.6 | - | | - | | - |  | 10.00 | 24.2 | 24.1 |
| 3.00 | 24.5 | 24.5 | - | | - | | - |  | 3.00 | 26.1 | 26.3 |
| 1.00 | 26.5 | 26.5 | - | | - | | - |  | 1.00 | 29.5 | 29.5 |
| 0.30 | 28.8 | 28.9 | - | | - | | - |  | 0.30 | 30.9 | 30.9 |
| 0.10 | 30.8 | 30.8 | - | | - | | - |  | 0.10 | 32.9 | 33.0 |
| 0.030 | 29.4 | 29.3 | - | | - | | - |  | 0.030 | 35.2 | 35.1 |
| 0.010 | 31.3 | 31.1 | - | | - | | - |  | 0.010 | 37.9 | 37.2 |
| 0.0030 | 34.8 | 33.2 | - | | - | | - |  | 0.0030 | 38.0 | 38.7 |
| 0.0010 | 35.7 | 34.6 | - | | - | | - |  | 0.0010 | - | 38.6 |
